# Downregulated XBP-1 Rescues Cerebral Ischemia/Reperfusion Injury-Induced Pyroptosis via the NLRP3/Caspase-1/GSDMD Axis

**DOI:** 10.1155/2022/8007078

**Published:** 2022-04-21

**Authors:** Yueting Zhang, Zhihui Yao, Yan Xiao, Xiaoling Zhang, Jiaxin Liu

**Affiliations:** ^1^The Second Affiliated Hospital of Kunming Medical University, Kunming, Yunnan 650100, China; ^2^926 Hospital of People's Liberation Army, Medical School of Kunming University of Science and Technology, Kunming, Yunnan 650500, China; ^3^Medical School of Kunming University of Science and Technology, Kunming, Yunnan 650500, China

## Abstract

Ischemic stroke is a major condition that remains extremely problematic to treat. A cerebral reperfusion injury becomes apparent after an ischemic accident when reoxygenation of the afflicted area produces pathological side effects that are different than those induced by the initial oxygen and nutrient deprivation insult. Pyroptosis is a form of lytic programmed cell death that is distinct from apoptosis, which is initiated by inflammasomes and depends on the activation of Caspase-1. Then, Caspase-1 mobilizes the N-domain of gasdermin D (GSDMD), resulting in the release of cytokines, such as interleukin-1*β* (IL-1*β*) and interleukin-18 (IL-18). X-box binding protein l (XBP-1) is activated under endoplasmic reticulum (ER) stress to form an important transcription factor XBP-1 splicing (XBP-1s). The cerebral ischemia/reperfusion (CI/R) causes cytotoxicity, which correlates with the activation of splicing XBP-1 mRNA and NLRP3 (NOD-, LRR-, and pyrin domain-containing 3) inflammasomes, along with increases in the expression and secretion of proinflammatory cytokines and upregulation of pyroptosis-related genes in HT22 cells and in the middle cerebral artery occlusion (MCAO) rat model. However, whether XBP-1 plays a role in regulating pyroptosis involved in CI/R is still unknown. Our present study showed that behavior deficits, cerebral ischemic lesions, and neuronal death resulted from CI/R. CI/R increased the mRNA level of XBP-1s, NLRP3, IL-1*β*, and IL-18 and the expressions of XBP-1s, NLRP3, Caspase-1, GSDMD-N, IL-1*β*, and IL-18. We further repeated this process in HT22 cells and C8-B4 cells and found that OGD/R decreased cell viability and increased LDH release, XBP-1s, NLRP3, Caspase-1, GSDMD-N, IL-1*β*, IL-18, and especially the ratio of pyroptosis, which were reversed by Z-YVAD-FMK and downregulated XBP-1. Our results suggest that downregulated XBP-1 inhibited pyroptosis through the classical NLRP3/Caspase-1/GSDMD pathway to protect the neurons.

## 1. Introduction

Stroke is a leading cause of permanent disability and death that affects about 15 million people around the world [1]. It is characterized by high rates of incidence, disability, and recurrence. Ischemic stroke is caused by the interruption of cerebral blood flow or the obstruction of the cerebral vasculature by thrombus, all of which deprives local brain tissues of oxygen and glucose [2, 3]. At present, pharmacological, physical, and modern mechanical reperfusion therapy is usually the first line of care in acute ischemia patients. In ischemic stroke therapy, reperfusion injury still is problematic [4]. Currently, reperfusion can elicit other life-threatening sequels that have prompted pursuance of studies involving the use of the cerebral ischemia/reperfusion (CI/R) animal model. This undertaking entails identifying novel targets to reduce the neuroinflammation induced by reoxygenation and glucose replenishment to revive compromised neural function. Neuroinflammation and neural cell injury in the infarct region are inescapable consequences of focal cerebral ischemia. Studies have shown that an inflammatory response not only goes along with the pathological development of CI/R but also is the primary cause of neuronal death [5]. Occlusion of the middle cerebral artery can bring about a prompt shutdown of the oxygen and glucose supply. Damage-associated molecular patterns (DAMPs) refer to oxygen-deprived cells in the ischemic region that upregulate and secrete factors such as TNF-*α*, interleukin-1*β*, interleukin-18, high mobility group box 1 (HMGB1), and extracellular cold-inducible RNA-binding protein (eCIRP). They can evoke dangerous signaling inflammatory mediators such as Caspase-1/Gasdermin, JAK/STAT-1, and TLR4/MyD88/NF-*κ*B [6].

An inflammasome is a type of a tissue-damage sensor that is necessary for the conversion of the proform of interleukin-1*β* (IL-1*β*) to the mature, active form and is also implicated with the pyroptosis [7–10]. Pyroptosis is distinct from apoptosis, which was first observed in macrophages infected with *Shigella flexneri*, intracellular bacteria [11] that enable the release of immunogenic cellular content, including DAMPs; these stressors activate NLRP3 (NOD-, LRR-, and pyrin domain-containing 3), and then, NLRP3 activates Caspase-1 by means of the adaptor apoptosis associated speck-like protein containing a CARD (ASC) [12]. Caspase-1 processes and activates inflammatory cytokines such as IL-1*β* and interleukin-18 (IL-18) and also cleaves gasdermin D (GSDMD) to release the membrane pore-forming N-terminal GSDMD domain (GSDMD-N). To trigger inflammation, GSDMD-N pores promote the release of activated IL-1*β* and IL-18 [13]. The NLR family, which includes NLRP3, IL-1*β*, and Caspase-1, has been reported to play critical roles in rodent models of the ischemic brain injury [14], and stroke is linked to a single-nucleotide polymorphism of IL-1*β* [15].

X-box binding protein l splicing (XBP-1s) serves as a significant transcription factor and is activated under endoplasmic reticulum (ER) stress, which can positively regulate both cell proliferation and angiogenesis. Previous studies have shown that transient cerebral ischemia can activate XBP-1 mRNA splicing to protect cells from ischemia/reperfusion-induced cell damage and apoptosis [16, 17]. XBP-1 can activate the NLRP3 inflammatory body [5]. XBP-1 deficiency in mice increases their susceptibility to both bacterial infections and impairs their host defense [18].

A recent study indicated that NLRP3 inflammasome deficiency or using its selective inhibitor (MCC950) can improve cerebral injury after ischemic stroke [19]. The IRE-1*α* inhibitor STF-083010 or genetic silencing of XBP-1 can selectively inhibit the IRE-1*α*/XBP-1s branch and then attenuate Cd-induced NLRP3 inflammasome activation and pyroptosis in HK-2 cells. Sirtuin-1 can ameliorate cadmium-induced ER stress and pyroptosis through XBP-1s deacetylation [20]. However, clear evidence that the increased expression and slicing of XBP-1 induced pyroptosis is present in cerebral ischemia/reperfusion has not yet been demonstrated. Accordingly, targeting XBP-1 and pyroptosis to suppress the abnormal inflammatory response may lead to a novel therapeutic strategy for CI/R injury [21].

In the present study, we explored the effects of XBP-1 on pyroptosis in HT22 cells, C8-B4 cells, and rats treated by OGD/R or middle cerebral artery occlusion (MCAO). We found that OGD/R decreased the cell viability, whereas and pyroptosis increased, which could be mostly inhibited by knockdown of XBP-1. OGD/R or MCAO increased the levels of XBP-1s, NLRP3, Caspase-1, GSDMD, IL-1*β*, and IL-18, which were restored by downregulated XBP-1 expression.

## 2. Materials and Methods

### 2.1. Materials

HT22 cells and C8-B4 cells were obtained from the Kunming Institute of Zoology (Kunming, China). Antibodies against NLRP3, GSDMD-N, IL-18, and XBP-1 were purchased from Abcam (Cambridge, MA, USA). Antibodies against XBP-1s and Cleaved Caspase-1 were purchased from Cell Signaling Technology (Massachusetts, USA). Antibody against Cleaved IL-1*β* was purchased from Affinity Biosciences (Jiangsu, China). Antibody against beta-actin was purchased form Proteintech Group, Inc. (USA). Z-YVAD-FMK was purchased from Selleckchem (Selleck Chemicals, China). Polyphyllin VI was purchased from MCE (MedChemExpress, USA).

### 2.2. Animals

Male Sprague-Dawley rats (weighing 250-280 g) were provided by the Shanghai Laboratory Animal Center, Chinese Academy of Sciences, and were housed under a under a 12 : 12 h light/dark cycle at 21 ± 1°C with ad libitum access to food and water. Animals were divided into four experimental groups: (i) sham group (sham), rats without carotid occlusion (S); (ii) I/R group, carotid artery occlusion was performed for 60 minutes followed by reperfusion for 24 hours (24 h); (iii) I/R group, carotid artery occlusion was performed for 60 minutes followed by reperfusion for 48 hours (48 h); and (iv) I/R group, carotid artery occlusion was performed for 60 minutes followed by reperfusion for 72 hours (72 h).

### 2.3. Middle Cerebral Artery Occlusion Model

We followed the methods of Deng et al. [22]. Rats were anesthetized with 1.5% isoflurane (Abbott, Abbott Park, IL, USA), and the left middle cerebral artery was occluded by the intraluminal suture technique as previously described (reversible middle cerebral artery occlusion without craniectomy in rats). Briefly, the middle cerebral artery was occluded by a 4-0 nylon monofilament coated with a silicone tip. Reperfusion was established by gently withdrawing the filament after 60 min of occlusion. In the sham control group, all of the surgical procedures were included except for the occlusion of the MCA.

### 2.4. Behavior Tests

#### 2.4.1. Gait Analysis

Gait was measured using the footprint test to assess limb coordination and stride length. For gait analyses, rats were habituated to the experimenter and the behavior room from 2 to 3 days before the test. Then, the rats were habituated to walk straight to their home cage. The paws of the rats were recorded by software (Bihaiwei Software Technology, Anhui, China); the rats were allowed to walk freely on the track. The mean stride length and mean stride width were analyzed by measuring the distance between paw prints.

#### 2.4.2. Neurological Assessment

We followed the methods of Li et al. [23]. Behavior testing is also critical for observing the degree of ischemia after 24 h, 48 h, and 72 h of reperfusion. Three blinded researchers rated and recorded the neurological deficit of the rats, and the scores of all the groups were then calculated. The 5-level 4-point Longa method was conducted in this study to evaluate the neurological deficit of each rat.

The criteria for scoring were as follows:

Grade 0: no neurological deficits

Grade 1: the contralateral forelimb cannot be stretched completely when the rat is lifted by its tail

Grade 2: the rat spontaneously circles to the paralytic side when walking

Grade 3: the rat involuntarily falls down to the contralateral side when walking

Grade 4: the rat cannot walk automatically and loses consciousness

#### 2.4.3. Evaluation of Infarct Volume

Two, three, five-triphenyltetrazolium chloride- (TTC-) stained brain sections were used to assess cerebral infarct volume. Twenty-four hours, forty-eight hours, and seventy-two hours after reperfusion, immediately after sacrifice, the brains were removed from the rats and cut coronally into five serial 2 mm slices. Samples were then incubated for 15 min in 2% TTC (Solarbio, Beijing, China) at 37°C and fixed in 4% paraformaldehyde overnight. Infarctions remained unstained by TTC. Each brain section of each rat was stained with TTC (unstained areas were recognized as infarctions) and was evaluated quantitatively using Image-Pro Plus software to calculate the percentage infarct volume.

#### 2.4.4. Haematoxylin-Eosin (H&E) Staining

Deeply anaesthetized rats were transcardially perfused with PBS followed by 4% paraformaldehyde. The brains were quickly removed by decapitation and carefully postfixed. Then, the samples were paraffinized and sliced to 5 *μ*m thick sections. The sections were dewaxed in 2 changes of xylene (10 min each) and rehydrated in 2 changes of absolute ethanol (5 min each) and then rinsed by running tap water orderly. Haematoxylin-eosin (H&E) staining was performed to observe the histomorphology. Histology assessment was performed by blinded investigator.

#### 2.4.5. Nissl Staining

For the Nissl staining, paraffin-embedded brain tissue sections (5 *μ*m) were immersed in xylene (5 min, 2 times), rehydrated in absolute ethanol (5 min, 2 times) followed by 95%, 75%, and 50% solutions of ethanol in water (5 min each), and then washed in distilled water for 2 times, 5 min each. Slides were stained in FD cresyl violet solution (FD Neurotechnologies, Baltimore, MD, USA) for 10 min and, then, briefly rinsed in 100% ethanol and differentiated in 100% ethanol containing 0.1% glacial acetic acid for 1 min. The slides were then dehydrated in absolute ethanol (2 min, 4 times) followed by clearance in xylene (3 min, 2 times). Coverslips were mounted with resinous mounting medium. The staining of the hippocampal CA1 region was routinely analyzed by a researcher blinded to the experimental protocol.

### 2.5. Cell Culture and Treatment with Drugs

HT22 cells (cell line) were cultured in F12 nutrient medium (Gibco, Carlsbad, CA, USA) supplemented with 10% fetal bovine serum (Gibco) and 0.5% penicillin–streptomycin (penicillin: 100 U/ml, streptomycin: 100 g/ml; Sigma-Aldrich, St. Louis, MO, USA), in a humidified atmosphere at 37°C under 5% CO_2_. C8-B4 cells (cell line) were cultured in DMEM nutrient medium, containing 25 mM glucose (Gibco, Carlsbad, CA, USA) supplemented with 200 mM glutamine (GlutaMax, Gibco), 10% fetal bovine serum (Gibco) and 0.5% penicillin–streptomycin (penicillin: 100 U/ml, streptomycin: 100 g/ml; Sigma-Aldrich, St. Louis, MO, USA), in a humidified atmosphere at 37°C under 5% CO_2_. Both cells were seeded in 6-well culture plate at a density of 4 × 104 per well. The cells were pretreated with Z-YVAD-FMK (20 *μ*M) for 30 min before stimulation with OGD/R. The cells were pretreated with polyphyllin VI (4 *μ*M) for 30 min before stimulation with OGD/R.

### 2.6. Transfections

We followed the methods of Bai et al. [24]. XBP-1 siRNA and negative control siRNA were chemically synthesized by Shanghai GeneChem Co., Ltd. (Shanghai, China). The sequences were as follows: XBP-1 siRNA, sense: 5′-CACCGGCTGCTCCAGCTCGCTCATC-3′; antisense: 5′-AAACGATGAGCGAGCTGGAGCAGCC-3′ and negative control siRNA, sense: 5′-UUCUCCGAACGUGUCACGUTT-3′; antisense: 5′-ACGUGACACGUUCGGAGAATT-3′.

HT22 cells and C8-B4 cells were seeded in 6-well plate at a density of 4 × 104 per well. The contents of 0.33 *μ*g siRNA and 5 *μ*l siTran transfection reagent (Origene, MD, USA) per well were diluted separately in serum free Opti MEM for a final volume of 250 *μ*l, gently mixed, and incubated for 5 min at room temperature. Then, the diluted siRNA solution and the diluted siTran transfection reagent were mixed gently and incubated for 20 min at room temperature. The diluted siRNA/siTran transfection reagent complex was added to the plates. After transfection with siRNA for 24 h, the cells were exposed to OGD/R and then harvested for assay.

### 2.7. Oxygen-Glucose Deprivation and Reoxygenation (OGD/R) Model

To mimic the CI/R conditions in vitro, the OGD/R modelling method was used, and the HT22 cells and C8-B4 cells were cultured under normal conditions for 24 h, then moved to glucose-free DMEM (Gibco), and placed under ischemic conditions (3% O_2_, 92% N_2_, and 5% CO_2_) at 37°C for 2 h. After that, the medium was discarded and the cells were cultured in normal medium under normoxic conditions for another 24 h reperfusion.

### 2.8. The Cell Viability Assay

We followed the methods of Bai et al. [24]. HT22 cells and C8-B4 cells were seeded into a 96-well plate overnight and then were pretreated with XBP-1 siRNA for 24 h and Z-YVAD-FMK (20 *μ*M) and polyphyllin VI (4 *μ*M) for 30 min before OGD/R and then were incubated for 24 h after exposure to OGD/R. The cell viability was measured by using the Cell Counting Kit-8 (CCK-8) according to the manufacturer's instructions. Twenty-four hours later, 10 *μ*l CCK-8 was added into every well, followed by 2 h 37°C incubation. Absorbance at 450 nm/630 nm was detected using an enzyme-labeled instrument. The results were obtained from three independent experiments, and each experiment was performed in triplicate. The mean OD of one group/mean OD of the control was used to calculate the viability.

### 2.9. Lactate Dehydrogenase (LDH) Assay

Cell death was evaluated by the quantification of plasma membrane damage which resulted in the release of lactate dehydrogenase (LDH). The level of LDH released in the cell culture supernatant was detected by LDH cytotoxicity assay detection kit (Beyotime, China) following the manufacturer's instructions.

### 2.10. Flow Cytometry (FCM)

HT22 cells and C8-B4 cells were washed with PBS (C0221A; Beyotime, Shanghai, China) and then digested with pancreatin (C0203; Beyotime, Shanghai, China) and next centrifuged at 168 × *g* for 5 min after OGD/R. The supernatant was discarded to collect the cells that were then resuspended in PBS. The cells in the suspension were counted and labeled with Annexin V (AV) and propidium iodide (PI) briefly, after which flow cytometry (Guava easyCyte™ 8, Millipore, USA) was employed to quantitatively determine and meticulously analyze the pyroptosis cells.

### 2.11. Western Blot Analysis

Protein lysates (rat tissues of hippocampus, HT22 cells, and C8-B4 cells) were prepared using a solubilizing solution (20 mM Tris HCl (pH 7.4), 150 mM NaCl, 1% NP-40, 1 mM EDTA, 1 mM PMSF, 1 mM EGTA, 1% TritonX-100, 2.5 mM sodium pyrophosphate, 1 mM Na_3_VO_4_, 1 mM beta-glycerolphosphate, 1 mg/ml leupeptinglycerolphosphate, and 1 mg/ml leupeptin). Protein concentration was determined by using Bio-Rad protein assay reagent (Hercules, CA, USA). An equal quantity of proteins was separated by SDS-PAGE of 10% for NLRP3 (1 : 1000) and 12% for GSDMD-N (1 : 1000), Cleaved IL-1*β* (1 : 500), IL-18 (1 : 1000), XBP-1s (1 : 1000), XBP-1 (1 : 2000), Cleaved Caspase-1 (1 : 1000), and *β*-actin (1 : 10000) and then transferred to PVDF membrane (Millipore Corporation, Billerica, MA, USA). The membrane was soaked in 5% skimmed milk (in PBS, pH 7.2, containing 0.1% Tween-20) overnight at 4°C and then incubated with primary antibody followed by peroxidase conjugated anti-mouse or anti-rabbit IgG (1 : 10000) (KPL, Gaithersburg, MD, USA). The epitope was visualized by an ECL Western blot detection kit (Millipore Corporation, Billerica, MA, USA). Other steps followed the instructions of each antibody. The detailed catalog numbers of all antibodies are listed in Table 1. Densitometry analysis was performed using ImageJ software.

### 2.12. Quantitative Real-Time Polymerase Chain Reaction (q-PCR)

We followed the methods of Bai et al. [24]. Rat tissues of hippocampus were harvested as described in the previous method. Total RNA was extracted from tissues using the RNAiso Plus reagent (TaKaRa, Japan, Cat. No. 108-95-2) according to the manufacturer's instructions. cDNA was synthesized using Revert Aid First stand cDNA Synthesis kit (ThermoFisher Scientific, UAB, K1622). To quantify the expression of Xbp1 spliced, Xbp1 unspliced, NLRP3, IL-1*β*, and IL-18, q-PCR was performed on a CFX96 Touch thermocycler (Applied Biosystems) using SYBR® Premix Ex Taq™ II (Applied Biosystems/ThermoFisher Scientific, UAB, A25742). Primer sequences for Xbp1 spliced, Xbp1 unspliced, NLRP3, IL-1*β*, IL-18, and GAPDH (Sangon Biotech, Shanghai, China) were used as follows in Table 2.

PCR amplification was carried out at 95°C for 30 s, followed by 45 cycles of 95°C for 5 s and 55°C for 30 s. GAPDH was used as an endogenous control to normalize differences. All fluorescence data were processed by a PCR postdata analysis software program. The differences in gene expression levels were analyzed with the 2^–*ΔΔ*CT^ method.

### 2.13. Statistical Analysis

Data were expressed as mean ± SEM. Statistical analysis was performed by using SPSS software. The one-way ANOVA followed by a post hoc Bonferroni multiple comparison test was used to compare control and treated groups. *p* value less than 0.05 was considered statistically significant. All blots are representative of experiments that were performed at least three times.

## 3. Results

### 3.1. CI/R Caused Behavior Deficits, Cerebral Ischemic Lesions, and Neuronal Death in the Rat MCAO Model

**Figure 1:**
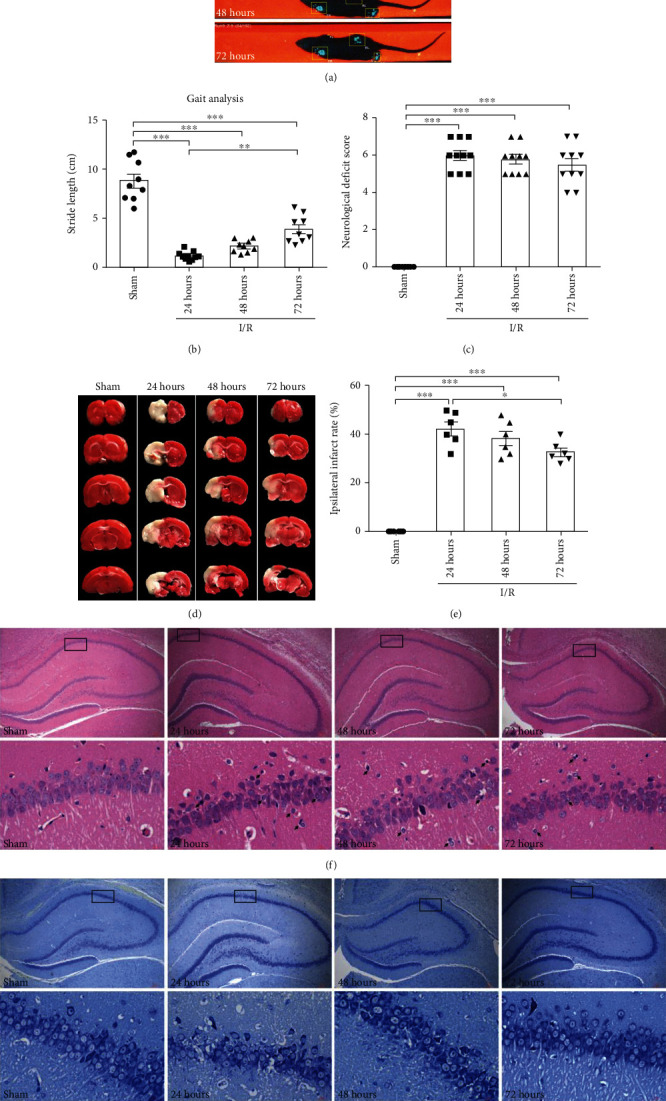
CI/R caused behavior deficits, cerebral ischemic lesion, and neuronal death in the rat MCAO model. Rats were treated with MCAO. (a) CI/R injured the gait of rats. Representative footprints of the rats are shown in each group. (b) The quantification of gait stride length (mm) (n = 9). (c) Neurological severity scores of each group evaluated after reperfusion (*n* = 9). (d) Cerebral ischemic lesion volume was detected by TTC, representative TTC-stained brain sections showing areas of healthy tissue (red) and ischemic injury (white) in different groups (*n* = 6). (e) Quantitative analysis of ischemic lesion volume (*n* = 6). (f) Haematoxylin-eosin (H&E) staining of the rat brain slices of each group in the hippocampal CA1 region (*n* = 3). (g) Nissl-stained sections indicated that C/IR increased neuronal death in the hippocampal CA1 region of rats (*n* = 3). Each bar represents the mean ± SEM. *p* > 0.05, ^∗^*p* < 0.05, ^∗∗^*p* < 0.01, and ^∗∗∗^*p* < 0.001 were statistically significant.

To confirm whether the rat MCAO model could successfully lead to CI/R injury, the gait analysis, neurological deficit score, and infract volume were evaluated in the sham group, 24 h group, 48 h group, and 72 h group. Compared to the sham group, the 24 h group, 48 h group, and 72 h group exhibited an abnormal gait (Figure 1(a)), and their stride length decreased significantly (Figure 1(b)) (*F*_(3, 32)_ = 60.3, *p* < 0.001). Meanwhile, the neurological deficit scores of the 24 h group, 48 h group, and 72 h group were significantly higher than that of the sham group (Figure 1(c)) (*F*_(3, 36)_ = 136.1, *p* < 0.001). TTC staining was used to evaluate the ipsilateral volume (Figure 1(d)). Compared to the sham group, the 24 h group, 48 h group, and 72 h group exhibited 42.3%, 38.3%, and 32.6% infarct rate, respectively (Figure 1(e)) (*F*_(3, 20)_ = 75.3, *p* < 0.001). To investigate the presence of CI/R-induced cerebral injury, the haematoxylin and eosin (H&E) staining and Nissl staining were used to observe changes in the model. The forms of neuronal death occurring in the hippocampal CA1 region of the 24 h group, 48 h group, and 72 h group were documented, including apoptotic cells (with membrane blebbing, shrunken soma, and concentrated nucleus) and pyroptotic cells (swollen, exhibiting a cabbage or fried-egg-like appearance with a detached nucleus in the center), while the sham group exhibited a normal appearance (Figure 1(f)). Compared to the sham group, an apparent decrease in the neuronal density in the CA1 region was noted in the 24 h group, 48 h group, and 72 h group (Figure 1(g)). These results demonstrated that the rat MCAO model was able to induce behavior deficits and neuronal death.

### 3.2. CI/R Activated XBP-1 Slicing, Neuroinflammation, and Neuron Pyroptosis in the Rat MCAO Model

**Figure 2:**
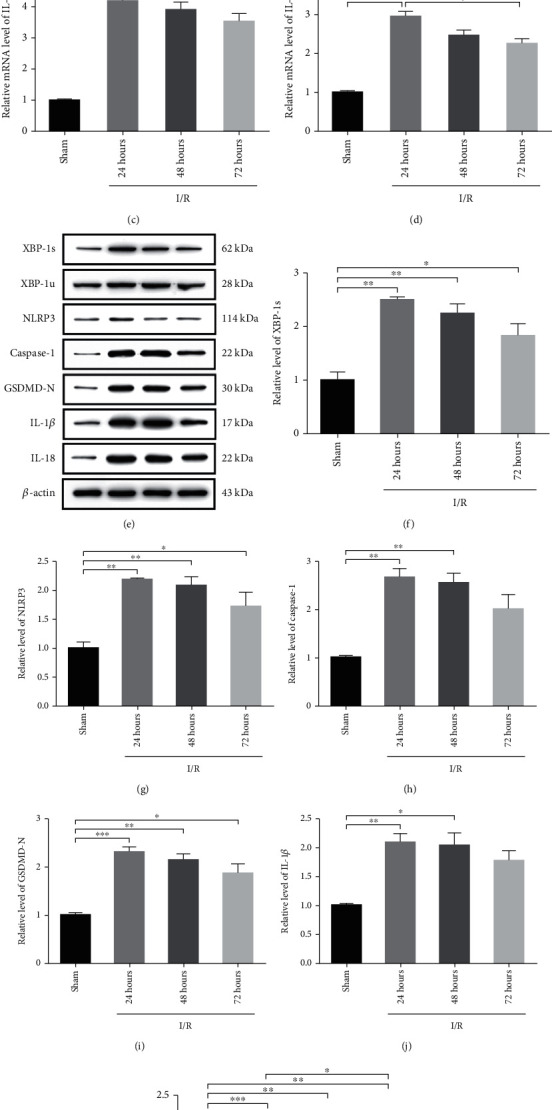
CI/R activated XBP-1 slicing, neuroinflammation, and neuron pyroptosis in the rat MCAO model. Rats were treated with MCAO. (a) The level of *Xbp-1s* mRNA in each group with CI/R. (b) The level of *NLRP3* mRNA in each group with CI/R. (c) The level of *IL-1β* mRNA in each group with CI/R. (d) The level of *IL-18* mRNA in each group with CI/R. (e) Representative western blots and quantification of the indicated proteins in the hippocampal lysate from rats in each group. Relative expression was normalized to sham rats. (f–k) The expression of XBP-1s, NLRP3, Caspase-1, GSDMD-N, IL-1*β*, and IL-18 in the hippocampus of the four groups after CI/R injury. Each bar represents the mean ± SEM (*n* = 6). *p* > 0.05, ^∗^*p* < 0.05, ^∗∗^*p* < 0.01, and ^∗∗∗^*p* < 0.001 were statistically significant.

Previous studies confirmed that XBP-1 is associated with CI/R, and the *Xbp-1s* mRNA expression level was measured. Compared to the sham group, the mRNA expression level of *Xbp-1s* was significantly increased in the 24 h group, 48 h group, and 72 h group (Figure 2(a)) (*F*_(3, 20)_ = 132.5, *p* < 0.001), and the expression of XBP-1s was also significantly increased in the 24 h group, 48 h group, and 72 h group (Figures 2(e) and 2(f)) (*F*_(3, 20)_ = 57.07, *p* < 0.001). XBP-1s can activate NLRP3 inflammatory bodies [20], and the signal of NLRP3 inflammasomes is a basic mechanism in CI/R. Compared to the sham group, the mRNA expression level of the *NLRP3* was significantly increased in the 24 h group, 48 h group, and 72 h group (Figure 2(b)) (*F*_(3, 20)_ = 164, *p* < 0.001), and the expression level of NLRP3 was also significantly increased in the 24 h group, 48 h group, and 72 h group (Figures 2(e) and 2(g)) (*F*_(3, 20)_ = 45.78, *p* < 0.001). The NLRP3 inflammasomes assemble and trigger the autocleavage of pro-Caspase-1 into Caspase-1 [25]. Compared to the sham group, the expression level of cleaved Caspase-1 was significantly increased in the 24 h group and the 48 h group (Figures 2(e) and 2(h)) (*F*_(3, 20)_ = 49.37, *p* < 0.001). Then, the active Caspase-1 generates an N-terminal GSDMD fragment (GSDMD-N) by cleaving GSDMD, which is recognized as a crucial executor of pyroptosis by inducing the formation of membrane pores [26]. Compared to the sham group, the expression level of GSDMD-N was significantly increased in the 24 h group, 48 h group, and 72 h group (Figures 2(e) and 2(i)) (*F*_(3, 20)_ = 63.88, *p* < 0.001). IL-1*β* and IL-18 were also evaluated and determined to be consistent with previous studies in which the mRNA expression levels of *IL-1β* (*F*_(3, 20)_ = 47.90, *p* < 0.001) and *IL-18* (*F*_(3, 20)_ = 47.99, *p* < 0.001), were significantly increased in the 24 h group, 48 h group, and 72 h group (Figures 2(c) and 2(d)). When compared to the sham group, the expressions of IL-1*β* (*F*_(3, 20)_ = 33.82, *p* < 0.001) and IL-18 (*F*_(3, 20)_ = 106.3, *p* < 0.001) were significantly increased in the 24 h group and 48 h group (Figures 2(e), 2(j), and 2(k)). These results suggest that CI/R injury can cause XBP-1 slicing, neuroinflammation, and neuron pyroptosis in the rat MCAO model.

### 3.3. OGD/R Decreased Cell Viability and Increased Cytotoxicity, XBP-1 Slicing, and Inflammation in HT22 Cells

**Figure 3:**
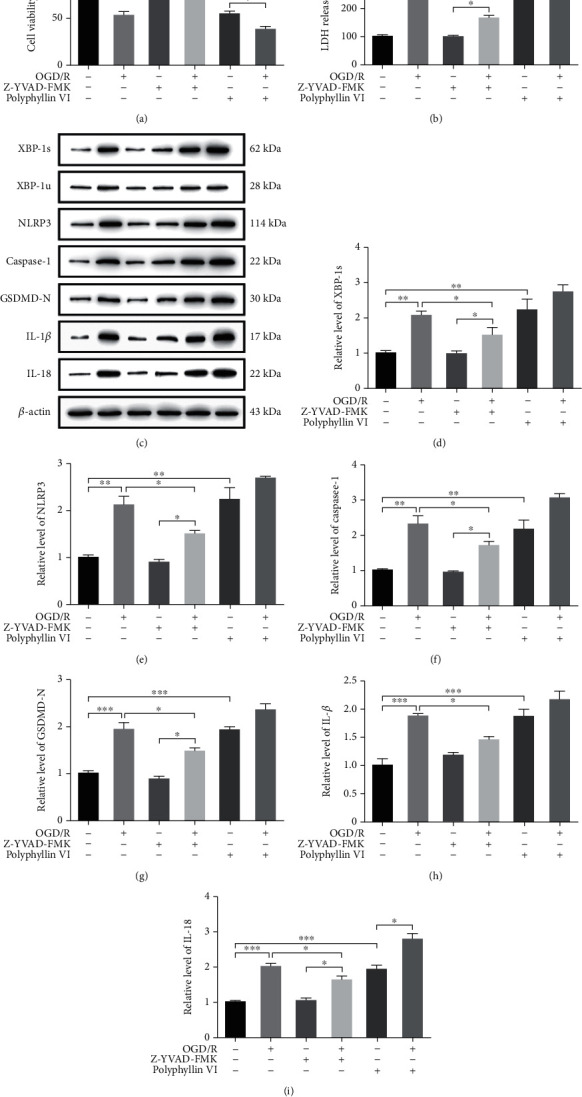
OGD/R decreased the cell viability and increased cytotoxicity, XBP-1 slicing, and inflammation in HT22 cells. The HT22 cells were, respectively, pretreated with Z-YVAD-FMK (20 *μ*M) and polyphyllin VI (4 *μ*M) 30 min before exposing to OGD/R for 24 h. (a) Cell viability was detected in HT22 cells using a CCK-8 assay. (b) LDH release was detected in HT22 cells using an LDH assay. (c) Representative western blots and quantification of the indicated proteins in the cellular lysate from HT22 cells in each group. Relative expression was normalized to that of the control group. (d–i) The expression of XBP-1s, NLRP3, Caspase-1, GSDMD-N, IL-1*β*, and IL-18 in HT22 cells after OGD/R. Asterisks indicate statistical significance (^∗^*p* < 0.05, ^∗∗^*p* < 0.01, ^∗∗∗^*p* < 0.001). All experiments were repeated for three times.

HT22 is a widely used hippocampal neuronal cell line. To investigate the effect of OGD/R on HT22 cells, the cellular viability and cytotoxicity were verified by using a CCK-8 assay and LDH release, respectively. As shown in Figures 3(a) and 3(b), OGD/R decreased the cell viability (*F*_(5, 12)_ = 91.9, *p* < 0.001) and showed obvious toxic effects (*F*_(5, 12)_ = 83.82, *p* < 0.001). To investigate which type of cell death was induced by OGD/R, Z-YVAD-FMK (the inhibitor of Caspase-1, 20 *μ*M) and polyphyllin VI (an inducer of Caspase-1-mediated pyroptosis, 4 *μ*M) were used as a positive control. In previous studies involving OGD/R and polyphyllin VI, cell death was induced, whereas the Caspase-1 inhibitor suppressed this response. OGD/R had similar effects to those of polyphyllin VI (Figures 3(a) and 3(b)). OGD/R increased the expression of slicing XBP-1 (*F*_(5, 12)_ = 14.57, *p* < 0.001), NLRP3 (*F*_(5, 12)_ = 29.12, *p* < 0.001), Caspase-1 (*F*_(5, 12)_ = 24.75, *p* < 0.001), GSDMD-N (*F*_(5, 12)_ = 35.33, *p* < 0.001), IL-1*β* (*F*_(5, 12)_ = 20.43, *p* < 0.001), and IL-18 (*F*_(5, 12)_ = 39.19, *p* < 0.001), which mostly could have been inhibited by Z-YVAD-FMK and was similar to the effects of polyphyllin VI (Figures 3(c)–3(i)). These results showed that OGD/R decreased cell viability, increased cytotoxicity, and induced XBP-1 slicing, inflammation, and pyroptosis in HT22 cells. XBP-1 may therefore be associated with pyroptosis in HT22 cells after OGD/R.

### 3.4. OGD/R Decreased Cell Viability and Increased Cytotoxicity, XBP-1 Slicing, and Inflammation in C8-B4 Cells

To confirm the above results in a different cell line, the murine C8-B4 microglia cell line was used. As shown in Figure S1A and S1B, OGD/R decreased the cell viability (*F*_(5, 12)_ = 48.22, *p* < 0.001) and increased the release of LDH (*F*_(5, 12)_ = 59.77, *p* < 0.001). OGD/R increased the expression of slicing XBP-1 (*F*_(5, 12)_ = 10.66, *p* < 0.001), NLRP3 (*F*_(5, 12)_ = 388.1, *p* < 0.001), Caspase-1 (*F*_(5, 12)_ = 95.57, *p* < 0.001), GSDMD-N (*F*_(5, 12)_ = 218.4, *p* < 0.001), IL-1*β* (*F*_(5, 12)_ = 20.62, *p* < 0.001), and IL-18 (*F*_(5, 12)_ = 27.05, *p* < 0.001), which mostly could have been inhibited by Z-YVAD-FMK and was similar to the effects of polyphyllin VI (Figure S1C–S1I). These results showed that OGD/R decreased the cell viability, increased LDH release, and induced XBP-1 slicing, inflammation, and pyroptosis in C8-B4 cells. Therefore, XBP-1 may be involved in pyroptosis in C8-B4 cells after OGD/R.

### 3.5. Downregulated XBP-1 Restored the Cell Viability and Inhibited the Cytotoxicity and Pyroptosis Induced by OGD/R in HT22 Cells

**Figure 4:**
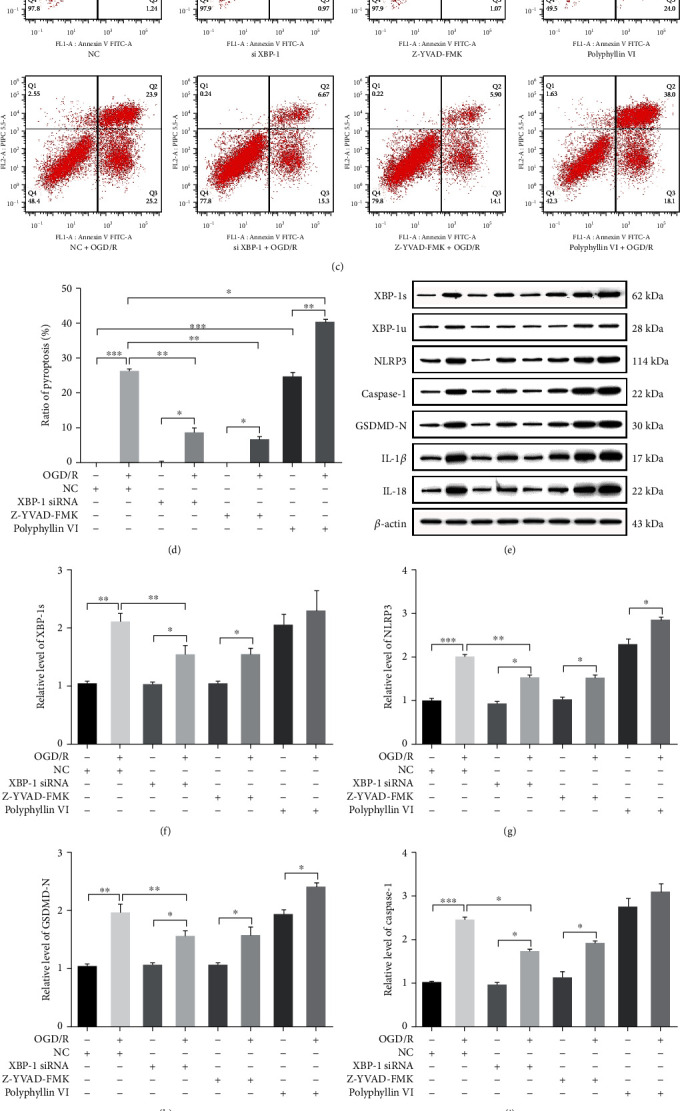
Downregulated XBP-1 restored the cell viability and inhibited cytotoxicity and pyroptosis by OGD/R in HT22 cells. The HT22 cells were, respectively, pretreated with XBP-1 siRNA, 24 h before exposing to OGD/R for 24 h and Z-YVAD-FMK (20 *μ*M) and polyphyllin VI (4 *μ*M) 30 min before exposing to OGD/R for 24 h. (a) Cell viability was detected in HT22 cells by using CCK-8 assay. (b) LDH release was detected in HT22 cells by using LDH assay. (c) The quantity of necrotic cells and pyroptotic cells were analyzed by Annexin V and PI staining in each group. (d) The ratio of pyroptosis was represented the percentage of Annv^+^/PI^+^ cell population. (e) Representative western blots and quantification of indicated proteins in the cellular lysate from HT22 cells in each group. Relative expression was normalized to control group. (f–k) The expression levels of XBP-1s, NLRP3, Caspase-1, GSDMD-N, IL-1*β*, and IL-18 in HT22 cells after OGD/R. Asterisks indicate statistical significance (^∗^*p* < 0.05, ^∗∗^*p* < 0.01, ^∗∗∗^*p* < 0.001). All experiments were repeated for three times.

To further examine whether XBP-1 expression can regulate Caspase-1-mediated pyroptosis, XBP-1 was downregulated using XBP-1 siRNA. As shown in Figures 4(a) and 4(b), OGD/R decreased the cell viability (*F*_(7, 16)_ = 53.14, *p* < 0.001) and increased LDH release (*F*_(7, 16)_ = 33.81, *p* < 0.001), which could have mostly been inhibited by downregulated XBP-1 in a similar manner to the effects of Z-YVAD-FMK. In this experiment, PI and Annexin V double staining were used to exhibit pyroptotic cells. The OGD/R group had a higher Annv/PI double-positive cell population (pyroptosis rate) than the control group (Figures 4(c) and 4(d)) (*F*_(7, 16)_ = 372.3, *p* < 0.001) and was the same as the polyphyllin VI group; however, downregulated XBP-1 can decrease the rate similarly to the effects of Z-YVAD-FMK. Downregulated XBP-1 also decreased the expression of slicing XBP-1 (*F*_(7, 16)_ = 10.42, *p* < 0.001), NLRP3 (*F*_(7, 16)_ = 140.4, *p* < 0.001), Caspase-1 (*F*_(7, 16)_ = 48.79, *p* < 0.001), GSDMD-N (*F*_(7, 16)_ = 35.67, *p* < 0.001), IL-1*β* (*F*_(7, 16)_ = 28.59, *p* < 0.001), and IL-18 (*F*_(7, 16)_ = 52.55, *p* < 0.001), like the effects of Z-YVAD-FMK (Figures 4(e)–4(k)). These results indicated that XBP-1 downregulation increased cell viability, decreased LDH release, and decreased XBP-1 slicing, inflammation, and pyroptosis in HT22 cells.

### 3.6. Downregulated XBP-1 Restored Cell Viability and Inhibited Cytotoxicity and Pyroptosis by OGD/R in C8-B4 Cells

We confirmed the above results in C8-B4 cells, as shown in Figure S2A and S2B; OGD/R decreased the cell viability and increased the release of LDH, which largely was inhibited by downregulated XBP-1, similar to the effects of Z-YVAD-FMK. The OGD/R group exhibited a higher pyroptosis rate than the control group (Figure S2C, S2D) (*F*_(7, 16)_ = 58.27, *p* < 0.001) and was the same as the polyphyllin VI group; downregulated XBP-1 may decrease the rate, in a similar manner as the effects of Z-YVAD-FMK. Downregulated XBP-1 also decreased the expression of slicing XBP-1 (*F*_(7, 16)_ = 46.63, *p* < 0.001), NLRP3 (*F*_(7, 16)_ = 31.18, *p* < 0.001), Caspase-1 (*F*_(7, 16)_ = 22.88, *p* < 0.001), GSDMD-N (*F*_(7, 16)_ = 24.5, *p* < 0.001), IL-1*β* (*F*_(7, 16)_ = 25.53, *p* < 0.001), and IL-18 (*F*_(7, 16)_ = 26.76, *p* < 0.001) similar to Z-YVAD-FMK (Figure S2E–S2K). These results showed that downregulation by XBP-1 increased the cell viability, decreased LDH release, and decreased XBP-1 slicing, inflammation, and pyroptosis in C8-B4 cells.

## 4. Discussion

**Figure 5:**
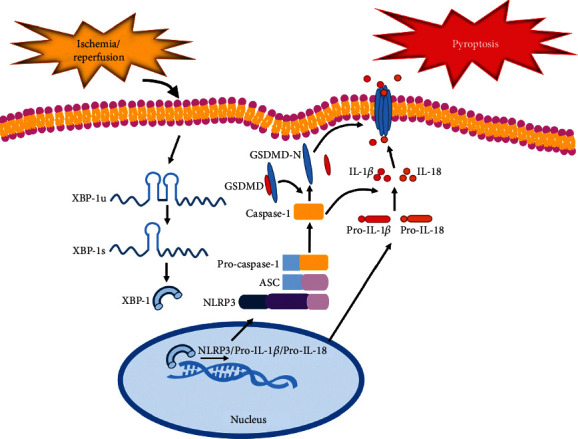
A schematic diagram for the mechanisms of XBP-1 in the regulation of neuronal pyroptosis following cerebral ischemia. The expression of XBP-1 is upregulated after cerebral ischemia/reperfusion. Cerebral ischemia/reperfusion mediates *Xbp-1u* mRNA splicing to generate the active *XBP-1s* form. Active XBP-1s promotes NLRP3-ASC/Caspase-1 inflammasome assembly, which generates inflammatory mediators and cytokines. The triggered Caspase-1 cleaves GSDMD to promote the release of the N-terminal domain, which executes pore formation on the neuronal membrane. The mature forms of IL-1*β* and IL-18 that are secreted through these pores are also increased. Downregulated XBP-1 can facilitate an anti-inflammatory effect and inhibit pyroptosis. XBP-1: X-box binding protein l splicing; NLRP3: NOD-, LRR-, and pyrin domain-containing 3; ASC: speck-like protein containing a CARD; GSDMD: gasdermin D; GSDMD-N: N-terminal GSDMD domain; IL-1*β*: interleukin-1*β*; IL-18: interleukin-18.

Ischemic stroke has become a major human threat, and the potential for reperfusion injury of cerebral ischemic tissue has attracted the attention of scientists [27]. Studies indicated that an inflammatory response is a major component of the pathological development of CI/R and is also one of the main causes of neuronal death [5]. In this study, we used an in vivo rat model and an in vitro cell culture model to investigate the potential mechanism of CI/R injury-induced neurotoxicity. Our results showed that pyroptosis mediated OGD/R-induced toxicity in both HT22 cells and C8-B4 cells. We also demonstrated that the reduction of XBP-1 mRNA by genetic means rescued OGD/R-induced activation of the NLRP3 inflammasome and pyroptosis through the NLRP3/Caspase-1/GSDMD pathway (Figure 5).

Unlike apoptosis, pyroptosis, which depends on the activation of Caspase-1 [28], is less understood in CI/R injury. The activation of Caspase-1 can lead to the cleavage of GSDMD [29] to generate the N-domain of GSDMD, which in turn forms membrane pores and mediates the release of cytokines, cellular swelling due to water influx, membrane rupture, and finally lysing the cell [23, 30–32]. Pyroptosis is characterized by the pore formation, osmotic swelling, and early loss of membrane integrity. By acting as a key pyroptotic executor, the GSDMD N-terminal fragment is able to induce the formation of membrane pores [26]. In this study, the inflammation-associated pyroptosis in both the rat MCAO model and the OGD/R cell model is described in detail (Figures 1–4 and Figure S1–2). H&E staining in the rat MCAO model (Figure 1(f)) revealed the characteristics of pyroptotic changes. These results suggest that pyroptosis is likely to be included in the responses induced by CI/R.

In microglia, active Caspase-1 can bring about pyroptosis by means of the intramembranous pores [33]. Driven by inflammasome activation, pyroptotic cell death in microglia exacerbated brain damage in ischemic stroke [34, 35]. Therefore, HT22 cells and C8-B4 cells were chosen to illuminate the mechanism of XBP-1 that influences pyroptosis. The NLRP3 inflammasome signal is the initial response that mediates the inflammatory response in the process of ischemic stroke [21]. In accordance with previous studies, our results confirmed that the NLRP3 inflammasome signal was activated within CI/R injury, and the mRNA level and expression of NLRP3 increased in the rat MCAO model (Figure 2). In addition, the expression of NLRP3 was increased by OGD/R (Figures 3 and 4), which was reversed by Z-YVAD-FMK.

The assembly of NLRP3 inflammasome triggers the pro-Caspase-1 autocleavage to active Caspase-1 [25], which is considered as regulating pyroptosis. Consistent with previous studies, this study indicated that pro-Caspase-1 was activated in CI/R injury; the expression of Caspase-1 was increased in the rat MCAO model (Figure 2). The expression of Caspase-1 was increased by OGD/R (Figures 3 and 4), which was reversed by Z-YVAD-FMK. Caspase-1 cleaves GSDMD to release the GSDMD-N domain and activates IL-1*β* and IL-18 [13]. Our results showed that the expression levels of GSDMD-N, IL-1*β*, and IL-18 were increased in the rats with CI/R injury (Figure 2), which was consistent with the rises in the mRNA expression levels of IL-1*β* and IL-18. In addition, the expression levels of GSDMD-N, IL-1*β*, and IL-18 were also increased by OGD/R (Figures 3 and 4) and were reversed by Z-YVAD-FMK. These results suggest that the inhibition of pyroptosis may prevent neuron damage by CI/R injury.

Polyphyllin VI can induce Caspase-1-mediated pyroptosis via the NLRP3/GSDMD signal axis [36]. In this study, polyphyllin VI was chosen as a positive control. OGD/R demonstrated the same capacity as polyphyllin VI for causing pyroptosis in both HT22 cells and C8-B4 cells (Figures 3–4 and Figure S1–2).

Increasing in vitro and in vivo evidence has demonstrated that CI/R injury activates ER stress. Upon unfolded protein response (UPR) activation, inositol-requiring transmembrane kinase/endonuclease-1 (IRE1) activates its endoribonuclease (RNase) activity through dimerization and autophosphorylation [37]. The IRE1 RNase removes a 26 nucleotide intron of the leucine zipper transcription factor XBP-1 and produces a frameshift in the XBP-1 mRNA transcript (the splicing mRNA of XBP-1). Then, the spliced XBP-1 (XBP-1s) mRNA is translated into a potent transcription factor that is responsible for the upregulation of genes encoding ER chaperones and proinflammatory gene production [38]. The activation of the XBP-1 serves to reestablish the homeostasis of the ER and the secretory pathway. Moreover, in addition to its importance in protein secretion and lipid metabolism, XBP-1s also modulates immune responses [37]. The transcription factor XBP-1 represents a key component of the ER stress response and is required for the maintained generation of proinflammatory cytokines during the inflammatory response. Previous researches have not clarified the relationship between XBP-1 and pyroptosis. In addition, previous studies indicate that deficient XBP-1 increases mice's susceptibility to bacterial infections and impaired host defenses [39]. Therefore, XBP-1 has a close relationship with NLRP3-associated inflammation and Caspase-1-mediated pyroptosis. Importantly, the inhibition of Caspase-1 activation using Z-YVAD-FMK or the knockdown of XBP-1 with siRNA substantially mitigated OGD/R-induced activation of the NLRP3 inflammasome and resulting cell death. Similarly, the IRE1/XBP-1 branch of the UPR signaling can regulate the inflammatory responses of macrophages. Using IRE-1*α* inhibitor, STF-083010 or genetic silencing of XBP-1 can selectively inhibit the IRE-1*α*/XBP-1s branch and then attenuate cadmium-induced NLRP3 inflammasome activation and pyroptosis in HK-2 cells [20]. Our results showed that XBP-1s was increased in the CI/R injury in both the rat model and the cellular model (Figure 2), and XBP-1 therefore may be a key factor. Downregulated XBP-1 can decrease the expressions of NLRP3, Caspase-1, GSDMD-N, IL-1*β*, and IL-18 (Figure 4 and Figure S2). Downregulated XBP-1 had the same effects as Z-YVAD-FMK. The decrease of IL-18 in particular has a meaningful influence on CI/R [40] in that it reduces the accumulation of macrophages and neutrophils and decreases the expression of proinflammatory molecules that are downstream of IL-18.

Our results showed that XBP-1 may be involved in pyroptosis and could play an important role in regulating pyroptosis in CI/R injury. Downregulated XBP-1 can inhibit NLRP3 inflammasome activation and potentially pyroptosis induced by OGD/R or MCAO. These effects may protect the neurons through NLRP3/Caspase-1/GSDMD pathway (Figure 5). These findings may offer a better understanding and novel therapeutic strategies for CI/R injury.

## 5. Conclusion

This study demonstrated that downregulated XBP-1 could inhibit pyroptosis by inhibiting the NLRP3/Caspase-1/GSDMD pathway in the hippocampus which rescues cerebral ischemia/reperfusion injury.

## Figures and Tables

**Table 1:** Antibody specifications.

Antibodies	Source	No.
NLRP3	Abcam	ab214185
GSDMD-N	Abcam	ab215203
Cleaved IL-1*β*	Affinity	#AF4006
IL-18	Abcam	ab243091
XBP-1s	Cell Signaling	#40435
XBP-1	Abcam	ab37152
Cleaved Caspase-1	Cell Signaling	#89332
Beta-actin	Proteintech Group	No. 66009-I-lg
Anti-rabbit IgG	KPL	No. 5450-0010
Anti-mouse IgG	KPL	No. 5450-0011

**Table 2:** RNA primer sequences.

Gene	Primer sequences
Xbp-1 spliced (forward primer)	5′-TCA GAC TAC GTG CGC CTC T-3′
Xbp-1 spliced (reverse primer)	5′-CTC TGG GGA AGG ACA TTT GA-3′
Xbp-1 unspliced (forward primer)	5′-CTG AGT CCG CAG CAG GTG-3′
Xbp-1 unspliced (reverse primer)	5′-CCA CAT CCG CCG TAA AAG AAT G-3′
NLRP3 (forward primer)	5′-CCA GAG CCT CAC TGA ACT GG-3′
NLRP3 (reverse primer)	5′-AGC ATT GAT GGG TCA GTC CG-3′
IL-1*β* (forward primer)	5′-CAG GCA GGC AGT ATC ACT CA-3′
IL-1*β* (reverse primer)	5′-AGG CCA CAG GTA TTT TGT CG-3′
IL-18 (forward primer)	5′-TCT TCA TTG ACC AAG GAA ATC GG-3′
IL-18 (reverse primer)	5′TCC GGG GTG CAT TAT CTC TAC-3′
GAPDH (forward primer)	5′-AGG TCG GTG TGA ACG GAT TTG-3′
GAPDH (reverse primer)	5′-GGG GTC GTT GAT GGC AAC A-3′

## Data Availability

All datasets presented in this study are included in the article. All data is real and guarantee the validity of experimental results.
