# Supplementary material for: Downregulated XBP-1 Rescues Cerebral Ischemia/Reperfusion Injury-Induced Pyroptosis via the NLRP3/Caspase-1/GSDMD Axis
Source: Mediators Inflamm. 2022 Apr 21;2022:8007078. doi: 10.1155/2022/8007078 (PMC9050284; doi:10.1155/2022/8007078)
Supplement: Supplementary Materials — Figure S1 and Figure S2 can be found in the Supplementary file. [file 8007078.f1.docx]

**FIGURE S1** OGD/R decreased the cell viability and increased cytotoxicity, XBP-1 slicing, and inflammation in C8-B4 cells. The C8-B4 cells were respectively pretreated with Z-YVAD-FMK (20 μM) and Polyphyllin VI (4μM) 30 min before exposing to OGD/R for 24 h. **(A)** Cell viability was detected in C8-B4 cells by using a CCK-8 assay. **(B)** LDH release was detected in C8-B4 cells by using an LDH assay. **(C)** Representative western blots and quantification of indicated proteins in the cellular lysate from C8-B4 cells in each group. Relative expression was normalized that of the control group. **(D-I)** The expression of XBP-1s, NLRP3, Caspase-1, GSDMD-N, IL-1β and IL-18 in C8-B4 cells after OGD/R. Asterisks indicate statistical significance (**p* < 0.05, ** *p* < 0.01, *** *p* < 0.001). All experiments were repeated for three times.

**FIGURE S2** Down-regulated XBP-1 restored the cell viability and inhibited cytotoxicity, and pyroptosis by OGD/R in C8-B4 cells. The C8-B4 cells were respectively pretreated with XBP-1 siRNA, 24 h before exposing to OGD/R for 24 h and Z-YVAD-FMK (20 μM) and Polyphyllin VI (4μM) 30 min before exposing to OGD/R for 24 h. **(A)** Cell viability was detected in C8-B4 cells by using a CCK-8 assay. **(B)** LDH release was detected in C8-B4 cells by using an LDH assay. **(C)** The quantity of necrotic cells and pyroptotic cells were analyzed by annexin V and PI staining in each group. **(D)** Ratio of pyroptosis. **(E)** Representative western blots and quantification of indicated proteins in the cellular lysate from C8-B4 cells in each group. Relative expression was normalized to that of the control group. **(F-K)** The expression of XBP-1s, NLRP3, Caspase-1, GSDMD-N, IL-1β and IL-18 in C8-B4 cells after OGD/R. Asterisks indicate statistical significance (**p* < 0.05, ** *p* < 0.01, *** *p* < 0.001). All experiments were repeated for three times.
